# Pixying Behavior: A Versatile Real-Time and *Post Hoc* Automated Optical Tracking Method for Freely Moving and Head Fixed Animals

**DOI:** 10.1523/ENEURO.0245-16.2017

**Published:** 2017-02-20

**Authors:** Mostafa A. Nashaat, Hatem Oraby, Laura Blanco Peña, Sina Dominiak, Matthew E. Larkum, Robert N. S. Sachdev

**Affiliations:** 1 Neurocure Cluster of Excellence, Humboldt Universität zu Berlin, Germany; 2 Berlin School of Mind and Brain, Humboldt Universität zu Berlin 10099, Germany; 3 Erasmus Program, Faculdad de Biologia, Universidad de Barcelona, Barcelona 08007, Spain

**Keywords:** behavioral analysis, closed-loop behavior, head-fixed behavior, real-time behavior, sensorimotor integration, whisker kinematics

## Abstract

Here, we describe an automated optical method for tracking animal behavior in both head-fixed and freely moving animals, in real time and offline. It takes advantage of an off-the-shelf camera system, the Pixy camera, designed as a fast vision sensor for robotics that uses a color-based filtering algorithm at 50 Hz to track objects. Using customized software, we demonstrate the versatility of our approach by first tracking the rostro-caudal motion of individual adjacent row (D1, D2) or arc whiskers (β, γ), or a single whisker and points on the whisker pad, in head-fixed mice performing a tactile task. Next, we acquired high-speed video and Pixy data simultaneously and applied the pixy-based real-time tracking to high-speed video data. With this approach, we expand the temporal resolution of the Pixy camera and track motion (*post hoc*) at the limit of high-speed video frame rates. Finally, we show that this system is flexible: it can be used to track individual whisker or limb position without any sophisticated object tracking algorithm, it can be used in many lighting conditions including infrared (IR); it can be used to track head rotation and location of multiple animals simultaneously. Our system makes behavioral monitoring possible in virtually any biological setting.

## Significance Statement

We developed a method for tracking the motion of whiskers, limbs, and whole animals in real time. We show how to use a plug and play Pixy camera to monitor the motion of multiple-colored objects in real time and *post hoc*. Our method has major advantages over currently available methods: we can track the motion of multiple adjacent whiskers in real time at 50 Hz and apply the same methods *post hoc* at a high temporal resolution. Our method is flexible; it can track objects with similar shape like two adjacent whiskers, forepaws, or even two freely moving animals. With this method, it becomes possible to use the phase of movement of particular whiskers or a limb to perform closed-loop experiments.

## Introduction

A traditional approach to the study of neural function is to relate activity of a circuit to a distinct behavior. While methods for measuring and manipulating neural activity have become increasingly sophisticated, the ability to monitor and manipulate behavior in real time has not kept pace. Even today, despite the advancement in the methods developed to precisely track animal behavior such as eye movement or head direction of animal in real time at different contexts (Hölscher et al., 2005; Wallace et al., 2013), in some of the most sophisticated closed-loop behavioral electrophysiology and imaging systems, i.e., visual virtual reality, where motion of the treadmill or air-ball is used to remap the visual world, there is no direct report of the animal movement; the motion of the animal is tracked indirectly by monitoring the movement of the treadmill or the air-ball (Legg and Lambert, 1990; Dombeck et al., 2007; Harvey et al., 2009; Cushman et al., 2013).

To overcome these kinds of limitations in behavioral monitoring, we used the whisker system, a model sensory motor system in which many of the key advances in monitoring neural activity *in vivo* have been used, i.e., calcium imaging of neurons and dendrites *in vivo*, imaging activity of axons, whole-cell patching in behaving animals, etc. (Svoboda et al., 1997; Svoboda et al., 1999; Lee et al., 2006; Gentet et al., 2010; Petreanu et al., 2012). While the whisker to barrel cortex system is a model for investigations of sensory motor processes, it has one key limitation; whiskers are tiny and can be difficult to track in real time. In the last decade, a variety of approaches have been used for monitoring whisker movement during behavior (Hires et al., 2013; Hentschke et al., 2006; Zuo et al., 2011; Sofroniew and Svoboda, 2015). High-speed videography is one common approach (Carvell and Simons, 1990; Sachdev et al., 2001; Hartmann et al., 2003; Knutsen et al., 2005; Ritt et al., 2008; Voigts et al., 2008; Grant et al., 2009; Clack et al., 2012; Arkley et al., 2014; Voigts et al., 2015). Another approach is to use electromyography (EMG) (Carvell et al., 1991; Fee et al., 1997; Berg and Kleinfeld, 2003; Sachdev et al., 2003; Zagha et al., 2013). Alternatively, a single or an array of laser/infrared beam break detectors was used for tracking the position of a whisker or the movement of the animal (Bermejo et al., 1996; O'Connor et al., 2013). Each of these approaches has advantages and disadvantages. EMG provides real-time feedback, but it does not have the spatial resolution for monitoring the motion of any individual whisker (Carvell et al., 1991; Fee et al., 1997; Berg and Kleinfeld, 2003; Sachdev et al., 2003; Zagha et al., 2013). High-speed imaging has unmatched spatial-temporal resolution; it can be used for monitoring one or multiple whiskers at a time, but it is typically not used in real-time or in feedback mode (Knutsen et al., 2005; O'Connor et al., 2010; Diamond et al., 2008; Voigts et al., 2008; Gyory et al., 2010; Perkon et al., 2011). In addition, automated tracking algorithms used with high-speed videography are often inflexible, as most tracking algorithms are customized to track a distinct object in a very specific setting. Most of the automated algorithms for tracking objects with high-speed cameras cannot track whiskers or limbs in systems where the floor and the walls around and under the animal move (Nashaat et al., 2016).

In this study, we present a method that turns an off-the-shelf camera (helped along by customized software) into a versatile real-time optical tracking system for monitoring whiskers, limbs, or whole animals. We can quantify the location, trajectory, and speed of almost any part of the body or of the whole animal. The same camera and algorithm can be used for offline tracking of movement, with almost no limit to the temporal resolution. This system makes it possible to analyze large quantities of video data and to generate continuous wave form of movement.

## Materials and Methods

### Animals

All animal procedures were performed in accordance with the animal care committee’s regulations. Mice were maintained in a reverse day/night cycle environment throughout the course of the experiments. Eight adult female mice were surgically prepared for head restraint by attaching a head-post to the skull under ketamine/xylazine anesthesia (90 mg/10 mg/kg). In the 2 d after surgery, Buprenex analgesia (0.1 mg/kg) was administered and the animal health was monitored. Rely-X cement was used to affix the head-post to the skull (Applicaps, 3 Com; Andermann et al., 2013). In two animals, a lightweight detachable Styrofoam color ID was affixed to the head-post to enable tracking of the freely moving animal.

**Figure 1.**
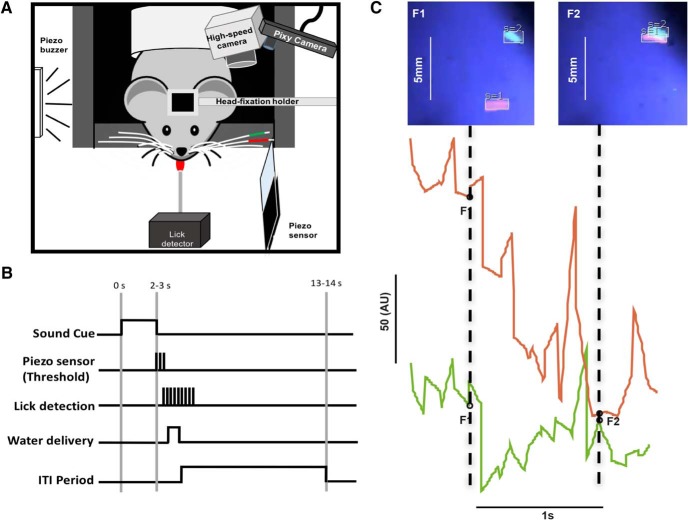
***A***, Setup design. Head-fixed mice are acclimatized to whisker painting and trained to use their whiskers to contact a piezo-film touch sensor. A Pixy camera was used to track whiskers in real time (left), a high-speed color camera was used simultaneously to acquire data. ***B***, Paradigm for whisker task. A sound-cue initiates the trial. The animal whisks one of the two painted whiskers into contact with a piezo-film sensor, and if contact reaches threshold, the animal obtains a liquid reward. There is a minimum inter-trial interval of 10 s. ***C***, Capturing whisker motion in real time. The movement and location of the D1 (green, S = 1, signature 1) and D2 (red, S = 2, signature 2) whiskers shown at two time points, frame 1 and frame 2 (below). The wave form of whisker data reflects the spatial location and the dimensions of the tracked box around the whisker. The waveforms in the middle show the movement of the two whiskers, toward and away from each other.

One to two weeks after surgery, animals were habituated to head-fixation on a stationary platform, or to head-fixation on a treadmill, or were allowed to explore a clear linear 42-cm-long × 9-cm-wide track made of Styrofoam. In subsequent days, animals were head-restrained for short periods of time, while individual whiskers were painted by dabbing Ultra-violet sensitive body paint (UV Glow) mixed with super glue. Mice were habituated to the coloring of whiskers and the placement of a piezo-film sensor at some fixed distance from the whiskers (Bermejo and Zeigler, 2000; Sachdev et al., 2001). Whisker contact with the sensor was rewarded with a drop of sweetened condensed milk. Mice were trained to move their whiskers in response to a sound cue (Fig. 1). Whisker contact of sufficient force against the piezo-film sensor elicited a reward (Fig. 1*B*). In the second task, animals were habituated to head-fixation while on a treadmill. The forepaws were painted with two different UV dyes one for each paw. For freely moving animals, a piece of multicolored Styrofoam (different colors combination for each animals) was glued to head-post and used for tracking mice in regular light conditions. In all paradigms, animals were water restricted and weights were monitored daily and maintained at >85% body weight.

### Experimental setting

A Pixy camera (Charmed labs, Carnegie Mellon University) was equipped with a 10-30 mm f1.6 IR lens and connected to the USB port of a computer. Pixy uses an HSV (hue, saturation, and value) color-based filtering algorithm to track colored objects. The open-source camera software, PixyMon, was used to mark up the colored whiskers and limbs defining a distinct signature for each color. Color signatures were tuned to achieve consistent tracking without generating false positives (detecting wrong objects) or false negatives (detecting the object intermittently or sparsely).

### Tracking software and importing data

**Figure 2.**
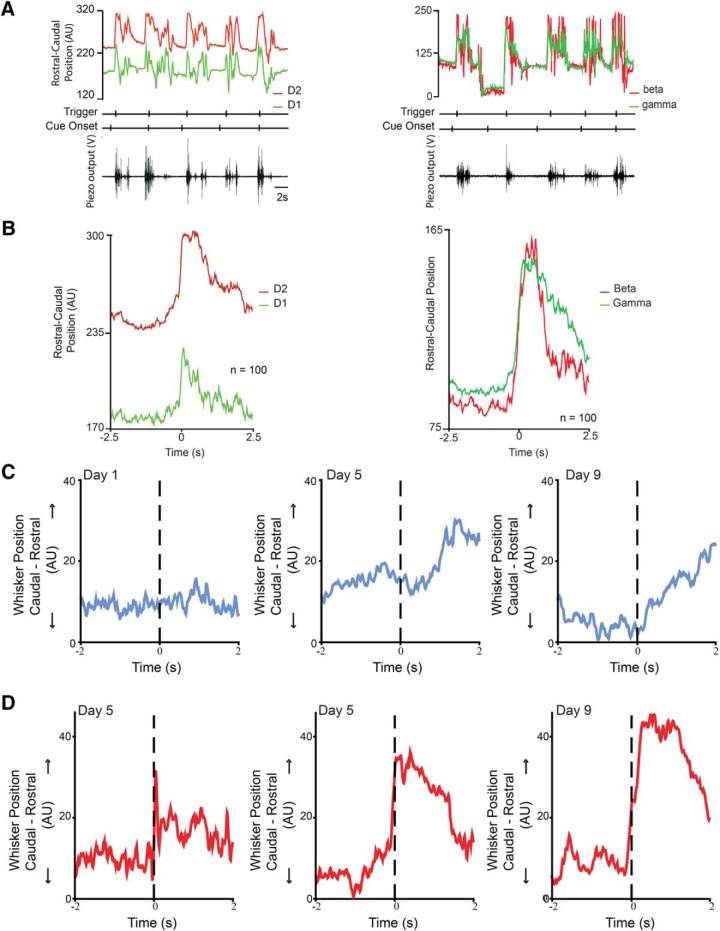
A real-time automated multiple whiskers tracking. ***A***, Pixy data from D1 and D2 whiskers (left, raw and smoothed) or β and γ whiskers (right, smoothed), as a mouse performs five auditory go-cue-triggered trials. A mouse moves a whisker into contact with a piezo-film sensor (bottom). Contact with the sensor triggers a reward. The cue onset and the reward trigger times are marked below the whiskers movement traces. Note that the spatial location of the D1 and D2 whiskers is distinct; the position of the two whiskers rarely overlap. In these trials, the distance between the two whiskers ranged from approximately 2 to 10 mm (distances converted into arbitrary units that denote spatial location). ***B***, Average position during task performance. The D1 and D2 whiskers move differently (left): the average position of the two whiskers at rest is different (before zero), and the average position of the two whiskers at contact is different (at zero). The D2 whisker, which contacts the piezo-film sensor and is rostral to the D1 whisker, moves more than the D1 whisker. In contrast, the two arc whiskers’ position overlaps at rest and at contact, but even here, the average motion of the whisker used to make contact with the sensor is different from the motion of the adjacent whisker. ***C***, Tracking performance by tracking whisker movement over days. The performance of an animal trained in the go cue task was monitored by monitoring the motion of the B2 whisker over days of training. The go-cue-triggered motion of the B2 whisker is task related by d 9 of training (compared with the imperceptible motion of the same whisker after the cue on d 1). ***D***, The contact-triggered motion is also faster and larger by d 9, compared with its motion on d 1 (on the left).

**Figure 3.**
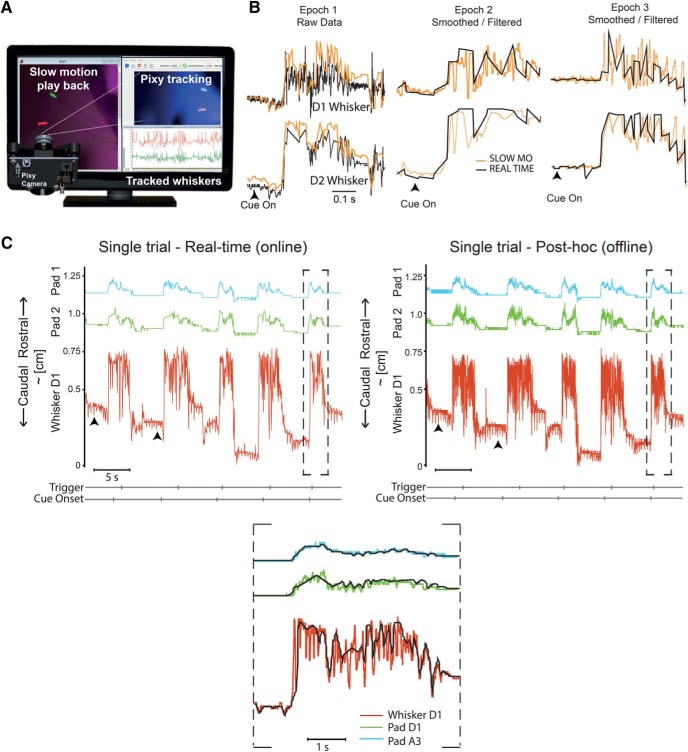
Post-hoc automated tracking. ***A***, Diagram of a Pixy camera capturing whiskers motion previously recorded with a high-speed video camera and played back in slow motion on a monitor. ***B***, Comparison of the high-fidelity signature of the D1 and D2 whiskers (top and bottom), recaptured automatically by the Pixy camera in slow motion (orange) with the data acquired in real time (black). ***C***, Motion of the two points on the whisker pad and one whisker are tracked in real time and *post hoc* in slow motion. The motion of the D1 whisker and the pad-point under the D1 whisker, and the second pad point under the A2 whisker could be tracked easily in real time and the same trials could be examined *post hoc* with analysis of the slow-motion playback of high-speed video data. The motion of the whisker pad appears to be a filtered version of the whisker motion. The motion of the D1 whisker in both real time (left) and *post hoc* (right) reveals differences in the set-point of protraction on each of the five trials, but real-time pixy data captures the entire envelope of both the whisker and the pad motion (bottom, expanded record of trial above on right).

**Figure 4.**
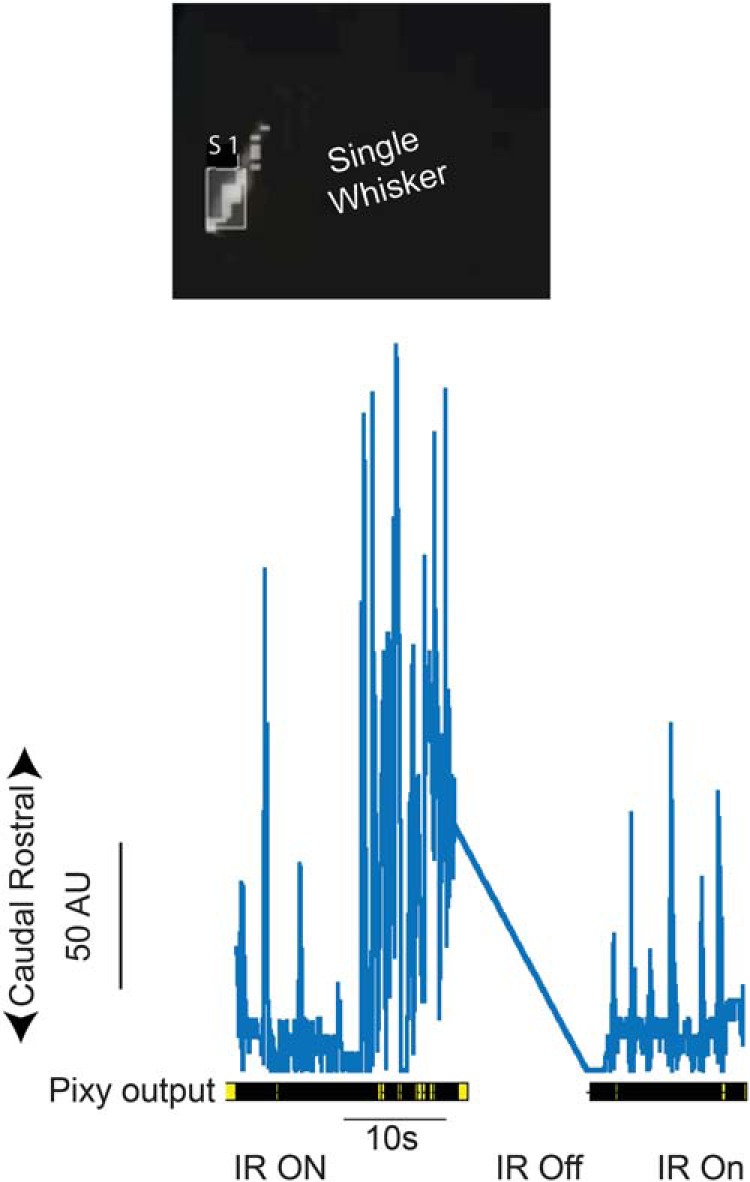
Single whisker tracking in infrared light. Top, Pixy image of whisker painted with yellow UV light sensitive paint, illuminated with infrared light only and automatically tracked in real time. Bottom, Output from Pixy camera showing periods with infrared (IR ON) and without infrared (IR OFF) illumination.

**Figure 5.**
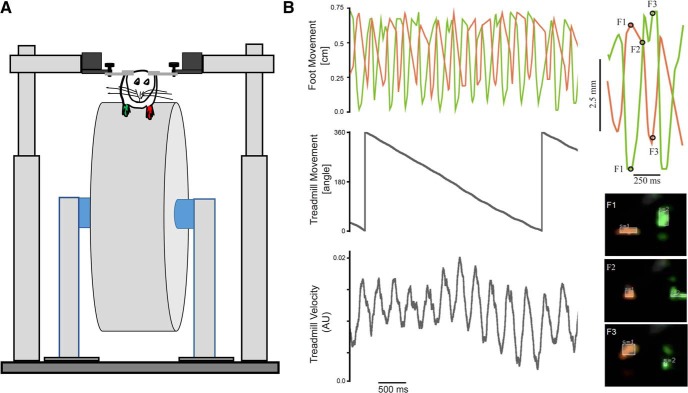
Pixy tracking of two limbs. **A**, schematic of a mouse head-fixed on a treadmill and the two forepaws, one painted green, the other painted red are tracked with a Pixy camera. **B**, The positon and velocity of the treadmill and the alternating up and down motion of the limbs are tracked in real time.

**Figure 6.**
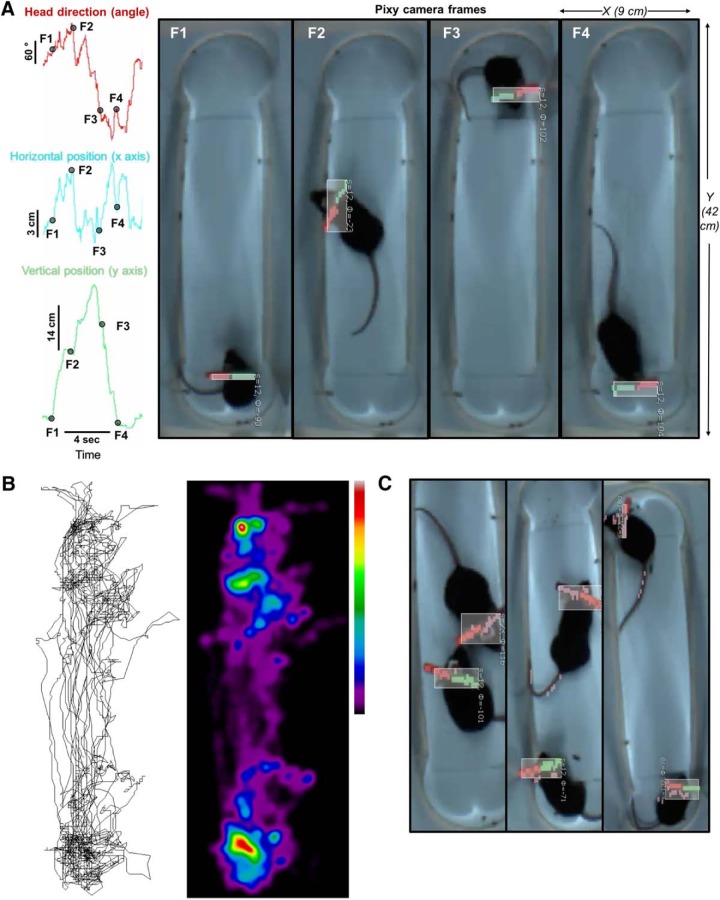
Tracking head rotation and location of freely moving animals. ***A***, The head rotation (top), *x* (middle), and *y* (bottom) coordinates of animal position were simultaneously tracked. Four time points corresponding to the four frames (right) are shown, where the animals head direction, and position in the box change from moment to moment. ***B***, The animal’s position over 3 min was tracked and a heat map of the preferred location was created; red, more time and blue, less time. ***C***, The location of two animals in the same enclosure can be distinctly tracked, including each animals head rotation, and position. Pixy tracking is shown by the boxes around the animal’s head.

PixyMon is the commercial computer software used to communicate with the Pixy camera. It is written using Qt language, which is an event-based C++ cross-platform framework widely used in GUI applications. PixyMon enables signature tuning, i.e., tuning the tracking of a colored object, via its configure dialogue tab. The tolerance of each signature can be optimized by adjusting a set of graphical sliders. The camera can learn up to seven distinct colors counting from “signature 1” up to “signature 7.” The user can either assign a signature as a “standard” signature where objects are detected based on a single color, or the user can assign a “color-code” signature in which detected objects consist of two or more adjacent colors in distinct sequence. The color-code signatures reduce false positives, as they limit the possibility that colors are confused with other similar objects in the camera view. In the color-code mode, PixyMon software reports the angle based on the position and rotation of two or more adjacent color. Here, we used the standard mode for tracking whiskers, the whisker pad, and limbs (Figs. 1-5) and use the color code for tracking the head rotation and location of the freely moving animal (Fig. 6).

### Signature-mapper

We modified PixyMon to send coordinates over the network using user datagram protocol (UDP) to a new software that we have developed and called the signature-mapper. This software can receive coordinates from multiple simultaneously running instances of PixyMon. It can also be used to automatically compress the video data played back in slow motion uniformly after acquisition with high-speed camera.

The signature-mapper is linked via a serial port to Spike2 (or it can be linked to MATLAB or another python application via UDP or transmission control protocol), or to a file to be stored on disk. In its current implementation, the signature-mapper allows seven different output channels (from C1 to C7). The source code and the binaries for the modified PixyMon and the signature-mapper are available at: http://www.neuro-airtrack.com/pixy_paper/pixy.html, https://github.com/larkum-lab, RRID: SCR_014813.

### System validation

The Pixy camera has a 50-Hz temporal resolution in real time. To measure the actual temporal resolution and delay from the Pixy camera to Arduino or Spike2/CED Power 1401 interface, we triggered a green LED with a TTL and turned it off at the first report of a signal from the camera. We recorded the timestamps of both LED trigger and the first serial message that reported that the LED turned on, from Pixy camera either directly through Arduino or indirectly through Pixy USB port connected to the PixyMon which sends the data to Spike2 via the signature-mapper software. We found that the time lag between triggering of the LED and reporting is ∼30 ms. In another test of the system, we used a colored object attached to rotary motor, where the frequency of movement could be altered between 5 and 20 Hz. This experiment showed that Pixy can be used to make complete wave form of motion at ∼8 Hz.

During whisker tracking in real time, there was a potential for false positives, or a false negative (missed frames). False positive frames usually develop when a colored object, a single painted whisker that can be reported as more than one signature (because of the angle or position of the colored whisker relative to the Pixy camera) is seen in two locations in the same frame. We excluded any frame which had more than one value for the same signature. Normally, this error is evident during real-time data collection and can be corrected by changing the lighting or recoloring the whiskers/limbs or the head of the animal. To correct for missed frames (false negatives), we use offline tracking and data synchronization (Fig. 3; see below).

### Resampling high-speed videography and synchronization

Synchronizing data stream obtained by PixyMon from high-speed camera in slow motion depends on temporal resolution of high-speed camera and the replay speed of the movies in slow motion. The signature-mapper software uses the values of recorded and replayed frame rates to process the offline tracking data and to synchronize it with the real-time video. The experimenter inputs the rate by which the recorded video was slowed down while the software applies a simple mathematical formula to perform the compression for the data stream obtained offline to fit the real-time value of the video.

### Data acquisition

Painted whiskers or limbs or color ID on the animal head showed continuous tracking without saturation or breakdown. Pixy adapts to a variety of light conditions, including dark-ultraviolet, infrared, incandescent (reddish hue), or fluorescent (bluish hue) light. The white balance for each lighting condition is automatically adjusted as the Pixy powers on. When light conditions change, the white balance can be reset by unplugging the Pixy camera or by pressing the reset button for 2 s. In dark light, we use no more than three colors. In IR light, a whisker was painted with fluorescent dye and tracked using illumination from an infrared light source (Thorlabs). On the treadmill, the same methodology was applied for tracking forepaws (one color for each paw). For freely moving animals, we tracked the head direction using multicolor signatures, called a color code with which object position and angle can be automatically tracked. For offline tracking, a Basler high-speed color camera (model number acA1920-155) was used to capture images at 155 Hz. The high-speed camera recordings were played back in slow motion on a screen while the Pixy camera was setup to track the colored objects off the screen. From day to day, the coordinates (units) can vary because of the positioning of the camera, the precise zoom used on the camera, and the angle of the camera. In the case of the β and γ whiskers, which are arc whiskers, there is considerable overlap in the position of the whiskers relative to the camera (Fig. 2).

Here, we use Spike2 (Cambridge Electronic Design) for data acquisition. A Spike2 script is used to transform the *x*, *y*, and angle text coordinates into waveforms. The Spike2 script is available online at: http://www.neuro-airtrack.com/pixy_paper/pixy.html, https://github.com/larkum-lab, RRID: SCR_014813.

### Data analysis

The real-time data from Pixy was mapped to Spike2 channels. When combined with the timing of behavioral events it is possible to take single trial (touch triggered or go-cue triggered) data for two adjacent whiskers and to make average waveforms for all movement data for each whisker over multiple trials. To show that both the *x* and *y* coordinates could be monitored by Pixy, we sampled the *x* and *y* coordinates of limb position and mapped this to Spike2 channels. In freely moving animals, the head rotation angle and *x*/*y* coordinates of animal position were acquired into Spike2 channels and converted into a linear track of movement of the animal or into heat maps of the animal. For the heat maps, we constructed a two-dimensional histogram of pixels in each video frame and applied 100 rounds of spatial filtering, where each pixel’s value was recomputed as the mean value of the pixel and each of its adjacent pixels (*n* = 8). Finally, high-speed video acquired at 150 Hz was played back at 6 Hz, and Pixy was used to capture the movement of whiskers into a Spike2 channel.

## Results

We used the Pixy-based system on head-fixed mice (*n* = 6). Five mice had their whiskers painted with UV-fluorescent paint and one mouse had both forelimbs painted (see Materials and Methods). A high-speed Basler camera and a Pixy camera were positioned to track two whiskers (Fig. 1*A*). In this paradigm, mice were conditioned to whisk to contact a piezo-film sensor after a sound go-cue turned on (Fig. 1*B*). To ensure that the painted whiskers were used in the contact task, the large whiskers rostral to the painted ones were trimmed off. We first determined whether the real-time whisker motion captured in video frames matched the position data recorded in real time (Fig. 1*C*). Video synchronized to the real-time data provided by Pixy indicated that both the absolute (real) and relative (*x* and *y* coordinates in the Pixy frame) whisker positions were tracked accurately (Fig. 1*C*, middle). In frame 1, the two painted whiskers are close to each other, in frame 2, both tracked whiskers are further apart. The total movement (in 20 ms) of the two whiskers is reflected in the length of the lines (Fig. 1*C*, middle), and the location of the red and green traces (lines) reflects the position of the whiskers in the two frames.

Next, we used these methods to track two adjacent whiskers (Fig. 2*A*, Video 1). The D2 and D1 or the β and γ whiskers were tracked in the course of five cue-triggered contacts. The mouse used the D2 or the β whisker to touch the piezo-film sensor. These five contact trials show that at rest and during contact with the piezo-film sensor, the position of D2 whisker rarely overlapped (<1 mm) with the D1 whisker (at least at the point where the two whiskers were painted). While the two whiskers position was distinct and nonoverlapping, the motion of the whiskers was in phase with each other. In contrast, when the arc whiskers (β and γ) were tracked (Fig. 2*A*, right), the whiskers showed considerable overlap in the rostro-caudal position. These data indicate that the spatial location of the whiskers can be accurately tracked. Next, we generated whisker touch-triggered averages of movement of the two painted whiskers in each animal (Fig. 2*B*). These experiments show that the whisker that touched the sensor (D2 or β) moved to a greater extent, i.e., there is a larger deviation from rest on average for the whisker used to elicit touch-triggered reward.

Video 1. Real-time tracking of D1 and D2 whiskers. Left panel shows the real-time data transmitted from Pixy to data files. The top right panel shows the simultaneously acquired high-speed video of the two whiskers, and the bottom right shows Pixy view. The D2 whisker is painted red and shows up as the red waveform on the top left, the D1 whisker is painted green and is the green waveform on the left. The yellow/black boxes are the text mark indicators, showing that Pixy is transmitting data in real time via the USB interface. The positions of the two whiskers do not overlap. They are not at the same point in space at the same time, in the videos or in the waveforms. The set point of both whiskers changes from moment to moment (time 5 s in the video, to 8 s in the video). The actual distance moved in millimeters can be seen in both the high-speed and the Pixy video. 10.1523/ENEURO.0245-16.2017.video.1

To examine whether we could use these methods to track the motion of a single whisker over days of training, we painted the B2 whisker each day and tracked the performance of a single mouse. On d 1 (Fig. 2*C*, left), the average sound cue-triggered whisker movement of the B2 whisker was minimal, but by d 9 of training, the B2 whisker moved immediately after the go-cue turned on (Fig. 2*C*, right). The whisker movement data for these days could also be aligned to the timing of contact; this also shows a change from d 1 to 9, in the average rate of movement, as the B2 whisker makes contact with the piezo-film (Fig. 2*D*).

The real-time temporal resolution of 50 Hz is borderline for the use of the Pixy camera for fast movements of the body, fast movements that include whisking, which in mice can reach 25 Hz. We therefore developed and validated another approach, an automated, offline, slow-motion approach using an additional high-speed video camera that is often used to faithfully track whisker motion. The recorded high-speed video behavior was played back on a computer monitor in slow-motion, and a Pixy camera was positioned in front of the monitor to track the colored whiskers (Fig. 3*A*, Video 2). For a fraction of cue-triggered trials, we compared the Pixy camera tracked slow-motion data to the simultaneously acquired real-time data (Fig. 3*B*). Surprisingly, the real time and the offline slow-motion waveforms are qualitatively similar, the position of the two whiskers (top traces are from one whisker bottom from another; Fig. 3*B*) does not overlap at rest or during contact, and the envelope and duration of movement of the adjacent whiskers looks similar in both conditions. In another experiment, we tracked two points on the whisker pad, one just under the D1 whisker and a second one under an A row whisker, and a single whisker, the D1 whisker in both real time and *post hoc* at 200 Hz (Fig. 3*C*). The five real-time and the five slow-motion epochs of the same trials shown here have a few elements that should be noted: (1) the protraction to contact begins at different positions on each of the five trials, and this is evident in both real time and *post hoc* slow-motion analysis; (2) pad motion does not quite capture the difference in set point from trial to trial; (3) whisker motion is evident when the animal is not whisking in both the real-time and slow-motion data (arrow heads point to deflection in the traces) but is clearer in the slow-motion data (Fig. 3*C*, right); and (4) the slow-motion data contains more high frequency components, but the envelope of motion is being captured in real-time and in slow-motion data (Fig. 3*B*,*C*, bottom). Taken together, this implies that for some purposes, the Pixy camera approach is appropriate. But the higher temporal resolution tracking of the offline video shows that the high frequency components of the movement are not captured in real time by the Pixy camera.

Video 2. Pixy analysis of slow-motion video data. The color high-speed video can be played back in slow motion (left panel). Pixy camera and Pixymon (middle panel) can be used to track the position of the two whiskers and the data can be extracted into a data file (right panel). 10.1523/ENEURO.0245-16.2017.video.2

To examine whether this method can be extended to infrared light condition (invisible to rodents), we painted a whisker with the same UV body paint, but instead of using UV dark light or regular illumination, we illuminated the whisker with infrared light. For proper IR illumination of just the whisker, the angle of the infrared light was key: the IR light was positioned under the Pixy camera and directed at the mouse whisker pad from the side. A single, painted whisker was tracked using a Pixy camera (Fig. 4, Video 3). Turning the infrared light off, removed all position information in the output. The text marks and the *y* position information were no longer generated and were no longer evident as a wave form. When the IR light was turned back on the real-time whisker, motion was reacquired and tracked without any additional adjustment.

Video 3. Pixy in infrared illumination. A single painted whisker shown in the video on the right is tracked in real time (left panel) with infrared illumination. At 3 s into the video, the infrared light is turned off, whisker color signature disappear as well. When the light is turned on again, the whisker can be tracked again. 10.1523/ENEURO.0245-16.2017.video.3

To demonstrate the flexibility of the Pixy camera system, we used it to track both forepaws of mice on a treadmill. The paws were painted with different colors, and the Pixy camera was positioned at the height of the forepaw of a mouse (Fig. 5, Video 4). In this configuration, we tracked the position of the treadmill, the velocity of the treadmill, and the up and down motion of each forepaw as the animal moved on the treadmill. Here, it is easy to see the alternating up and down motion of each limb as the animal moves forward on the treadmill.

Video 4. Tracking forepaws movements. The painted limbs can be tracked for alternating up and down movement in real-time. The red trace is for the UP/Down movement of the right limb. The green traces is for the left limb. The treadmill position and velocity are also shown in the traces below. 10.1523/ENEURO.0245-16.2017.video.4

Finally, we used Pixy to track head rotation and *x*/*y* coordinates of freely moving animals position in a 42 × 9 cm wide box in real time (Fig. 6*A*,*B*, Video 5). The moment by moment changes in head angle and animal location data (*x* and *y* coordinates) can be transformed into wave form (Fig. 6*A*) where F1 (related to the vertical position of the animal in frame 1 on the right) is at the bottom and has a value close to zero. In frame 1, the animals head angle is horizontal, in frame 2, the angle rotates by ∼70°, in frames 3 and 4, the angle is rotated by 180° (compared with frame 1; Fig. 6*A*). The side to side position of the animal changes, with the animal sometimes hugging the right side (frames 1, 3), the left side (frame 2) or is roughly in the middle of the box. The position of the animal can be traced at 50 Hz (Fig. 6*B*), and a heat map of the animal location in the box over 3 min of tracking can be constructed. In addition to tracking the location of individual animals, Pixy can be used to track multiple color IDs affixed to the animal head (Fig. 6*C*), thus simply and flexibly tracking one or multiple distinct freely moving animals.

Video 5. Tracking a single animal head rotation/direction and position in real time. Pixy camera tracks a multicolored piece of Styrofoam fixed on animal head-plate in regular light condition. The red traces on the top left shows the angle of head direction, while the blue traces in the middle left and green trace in bottom left shows the horizontal and vertical movement, respectively. 10.1523/ENEURO.0245-16.2017.video.5

## Discussion

This study demonstrates the utility of a color tracking camera that can be used for rapid real-time tracking of two adjacent whiskers, limbs, or even multiple animals. The method is flexible; it can work in various lighting conditions, and it can be used for real-time data acquisition and for automated tracking.

While earlier work in the whisker system has successfully used high-speed imaging, and EMG to detect motion of the whisker pad or of individual whiskers, these methods have limitations and advantages mentioned in the introduction. Aside from being easy to use and inexpensive, the Pixy method has key advantages over other methods (highlighted in Table 1**)**, foremost among them is that Pixy is versatile and can be used for tracking almost any colored object, one or multiple distinct whiskers, points on the whisker pad, limbs, or even whole animals, in real time. It is flexible enough to be rapidly reconfigured for monitoring any part of the body, multiple body parts, and even the whole animal. Furthermore, Pixy is an open-source tool, where almost every aspect of the process the data stream, the, PixyMon software, the objectives used, even the lighting, and coloring are accessible and modifiable.

**Table 1.** Comparison of videography, optoelectronic, and EMG methods to Pixy

		Bermejo et al. (1996)	Knutsen et al. (2005)	Voigts et al. (2015)	Ritt et al. (2008)	O'Connor et al. (2010)	Gyory et al. (2010)	Perkon et al. (2011)	EMG	Pixy (our method)
1	Tracking principle	Opto	Video	Video	Video	Video	Video	Video	Muscle	Opto/video
2	Spatial element	Single point	Multiple points	Multiple points	Multiple points	Multiple points	Multiple points	Multiple points	NA	Multiple points
3	Real time	Yes	No	No	No	No	No	No	Yes	Yes
4	Individual whisker	Yes	Yes	Yes	Yes	Yes	No	No	No	Yes
5	Number of single identifiablewhiskers	One whisker on each side	One whisker on each side	One whisker on each side	Three whiskers	Five whiskers	N/A	N/A	NA	Two whiskers (up to seven in principle)
6	Whisker removal	Yes	Yes	Yes	Yes	Yes	N/A	N/A	No	No
7	Limitation	Whisker thickness	Contrast andresolution	Contrast and resolution	Contrast and resolution	Contrast and resolution	Contrast and resolution	Contrast and resolution	NA	Illumination and color
8	Method shows	Single whisker	Single row	C1-4 whiskers	Single whiskers	Multiple whiskers	Two rows	Full whisker pad	Whisker pad	Two whiskers/one whisker and two pad/two paws
9	Head tracking	No	Yes	Yes	Yes	No	Yes	Yes	Yes	Yes
10	Head tracking requirement	N/A	Additional light source for the eye	Tip of nose	Contour edge/whisker base	N/A	No requirement	No requirement	Wire in muscle	Marker glued to head
11	Compatible in IR	Yes	Yes	Yes	Yes	Yes	Yes	Yes	Yes	Yes (single whisker)
12	Algorithm flexibility	Yes (not automatic)	No	No	No	No	No	No	Yes (with wires)	Yes
13	Unrestrained animal	No	Yes	Yes	Yes	No	Yes	Yes	Yes	Yes (whole animal)

Here we compare 13 different features of 7 earlier tracking methods, including optoelectronic (Opto), electromyography (EMG), and other whisker tracking algorithms combined with high-speed videography to our Pixy based method. The elements that we compared here: 1) Tracking principle: Tracking algorithms based on videography, optoelectronic methods like beam breaking, EMG or color. 2) Spatial coordinate system: Beam breaking has a distinct (single or multiple) spatial coordinate, while videography can track over multiple spatial locations. 3) Real-time at any frequency. 4, 5, 6) Number of objects tracked: A single whisker, or multiple individual whiskers, with or without plucking or removing whiskers. 7) Limiting element of each method: Lighting, contrast, resolution and length of whiskers for videography, or color and painting for Pixy, 8) Output: Single whisker, multiple whisker or whisker and whisker pad. 9, 10) Head direction tracking method: possibility, and whether the eye need or tip of the nose or a color needs to be tracked. 11) Ability to tack in infrared red light: All the high speed cameras can work with infrared light, as can EMG and optoelectronic methods. The pixy camera is limited in this context because it can only be used to track a single spatially distinct point with a pixy camera. 12) The flexibility in tracking multiple body parts: Cameras can be used for tracking any object, but optoelectronic methods, and EMGs, and even automated tracking video systems have to be optimized or positioned for tracking the object of interest. 13) The ability to use the system in unrestrained animals.

Most other methods are not nearly as flexible: videography is not commonly used in real time; EMG cannot be used for single whisker tracking; and optoelectronics, IR beam breaking methods, can be used only in designated locations (Table 1). Most earlier methods are not versatile enough and have not currently been used for any level of individual whisker or whisker-combined with whisker-pad tracking in real time. The Pixy approach has many advantages over other methods, but it also has some drawbacks. First, is that color is necessary and must be visible on the animal. Coloring, i.e., painting, adds some weight to a whisker and requires that the animal be habituated to the repeated application of body paint on animal’s limbs or whiskers. In addition, using a color-filtering algorithm limits the use of the system in infrared light, where Pixy can be used to track only one object. This limitation can be overcome by adding more than one Pixy camera to track each limb, or track a single whisker on each side of the face. Another limitation of the Pixy system is that it does not automatically provide a frame by frame update, rather it generates a serial time stamp of the tracked object. This limitation can be overcome by using TTL-triggered image capturing methods. Finally, another limitation is the temporal resolution of 50 Hz, where the actual resolution can be lower, depending on the configuration of the acquisition system. This temporal limit can be overcome *post hoc*. For studies where it is necessary to monitor higher frequency movement (>∼50 Hz), the Pixy camera can still be used to automatically track motion in slow-motion videos. A major element of this experimental design is that the fast movements missed in real time can be recaptured for analysis. Furthermore, key events (e.g. object contacts, etc.) can be still be tracked online using the Pixy camera during the behavior and can be used offline to quickly direct the researcher to important parts of the high-speed video images.

The advantage of the color-based system over the earlier automatic tracking software packages (Knutsen et al., 2005; Diamond et al., 2008; Voigts et al., 2008; Gyory et al., 2010; O'Connor et al., 2010; Perkon et al., 2011; Voigts et al., 2015) is that tracking depends on colors, where within some limits, the changes in lighting, the presence of motion under the whiskers, around the animal, and even changes in focus are less relevant than in most high-speed video imaging experiments. With the Pixy-based method, it becomes possible to noninvasively, flexibly, and inexpensively configure experiments where motion or location of one or more whiskers, limbs, or even the movement of the animal is used as feedback to trigger rewards, optogenetic signals, or even to change the real or virtual environment around the animal (Nashaat et al., 2016).

While our methods are by no means the first using color filtering, the range of tracking used in the work presented here, from tracking adjacent whiskers, to tracking freely moving animals, with little essential change in algorithm is unique and makes our methods almost universally applicable to a variety of settings and species (Bobrov et al., 2014; Cheung et al., 2014; Varga and Ritzmann, 2016).

## Synthesis

Reviewing Editor: Liset Menendez de la Prida, Instituto Cajal CSIC Decisions are customarily a result of the Reviewing Editor and the peer reviewers coming together and discussing their recommendations until a consensus is reached. When revisions are invited, a fact-based synthesis statement explaining their decision and outlining what is needed to prepare a revision will be listed below. The following reviewer(s) agreed to reveal their identity: Xavier Leinekugel, Mayank Mehta.

We all agree this is a novel approach for tracking and the ms is potentialy suitable for eNeuro. However we have all raised some concerns, and a detailed revision is required.

Apart from several details (references, labels in figures, etc..) we all have concerns regarding some methodological issues. You can see our three independent reports below, but basically you need to adress the following three points in your revision:

- Validation of the new system as compared to previous standards, which can be examined for the case of whiskers. Also, add reference to other tracking systems and discuss pros and cons of the Pixy approach

- Issues afecting potential synchronization between recording systems that are poorly considered in the current version. Describe how the Pixy is connected to the recording system, what is recorded and how different data streams are synchronized

- Concerns regarding the concept of real time and how synchronization between data streams could be affected when resampling from slow motion movies is implemented.

- Validate the quality of synchronisation and of detection (missed frames, variable lag between tracking/video stream and physiological data acquisition, false positives and negatives, etc...)

I hope these comments may be helpful to improve your ms.

Comments by R1:

The manuscript is describing the potential use of a commercial system, Pixy, for Neuroscience applications requiring the tracking of objects that can be distinguished by their colour(s). In particular, it shows that the Pixy system can be used for the tracking of small laboratory animals or any body part labeled with a specific colour tag. The product appears indeed useful. However, the system's web-page offers already a fair description of possible uses, with tutorials, videos and demos. The added value of the present paper compared to this already available information is limited. But providing detailed scripts or mounting schemes allowing easy implementation would be useful to the Neuroscience community. The manuscript reports the possible use of the commercial software Spike2 for tracking data acquisition from the Pixy camera, but without sufficient details to clearly know what is recorded (the video, the tracking information, or both), how and how precisely it is synchronized with the recording of physiological data, and how to proceed exactly to perform the recordings (e.g. how to connect the Pixy camera to the CED system). Therefore, this is a potentially useful method article if it can provide more detailed practical information on how to implement the combined recording of physiological and Pixy data, in particular regarding the question of synchronization and replay of the recorded information

Comments by R2:

This manuscript describes the Pixy camera based system for tracking whisker and paw movements of head fixed mice and head movement of freely behaving mice. Advantages of this system is that it is relatively noninvasive, inexpensive and off the shelf, and it can track many different aspects of movement.

This is an interesting method with good potential for neurophysiology. But, my enthusiasm is dampened for many reasons.

There is no clear validation or quantification of the new system. For example, what was the false positive or the false negative rate of whisker or paw tracking? What was the noise in these measurements? These issues are not even mentioned, let alone discussed.

Head position and head angle of freely behaving rats has been tracked at least since 1971 (e.g. O'Keefe & Dostrovky, 1971) and this is a standard feature of many commercial systems. Not only should studies cited, but the advantage, if any, of the pixie system over the existing methods of head position tracking discussed.

Line 47, references are incomplete. Ditto in many other places.

The list of citations is woefully inadequate. For example, papers on high speed videography of behaving rodents by Wallace et al. 2013, virtual reality systems that allow head movement and track head movement (Holscher et al. 2005, Cushman et al 2013).

The manuscript is written poorly, with little detail and too many repetitions

Comments by the AE:

- Introduction and discussion are biased towards the whisker system, but the method meant to be more general. Authors may try rebalance the focus.

- The concept of real time (abstract, fig.2) needs to be clarified. Fast behaviours require off line resampling with the camera from slow motion movies.

- Issues affecting appropiate synchronization between signal streams need to be addressed. This may be critical for closed-loop applications

- Check reference thourhgout the ms

- What is S=1, S=2 in Fig.1c?

- A curiosity: The camera is color-based. Are painted-super-glue whisker heavier? Can animals see them? I wonder whether this affects natural behaviour.
